# Cognitive Trajectories in Older Adults Diagnosed With Hematologic Malignant Neoplasms

**DOI:** 10.1001/jamanetworkopen.2024.31057

**Published:** 2024-08-30

**Authors:** Li-Wen Huang, Ying Shi, W. John Boscardin, Michael A. Steinman

**Affiliations:** 1 Division of Hematology/Oncology, Department of Medicine, San Francisco Veterans Affairs Medical Center, California; 2 Helen Diller Family Comprehensive Cancer Center, University of California San Francisco; 3 Division of Geriatrics, University of California San Francisco; 4 San Francisco Veterans Affairs Health Care System, San Francisco, California; 5 Department of Epidemiology and Biostatistics, University of California San Francisco

## Abstract

**Importance:**

More people are surviving long-term after diagnosis with hematologic malignant neoplasm (HMN), yet there are limited data on cancer-related cognitive impairment in people with HMN. Better understanding cognitive outcomes after HMN in older adults is important for patient counseling and management.

**Objective:**

To model cognitive trajectories and rates of cognitive decline before and after HMN diagnosis in older adults compared with a matched noncancer cohort.

**Design, Setting, and Participants:**

In this population-based cohort study, older adults from the Health and Retirement Study (HRS) diagnosed with HMN between 1998 and 2016 after age 65 years were matched 1:3 to participants without cancer from the same HRS wave using propensity scores incorporating variables relevant to cognition. Cognitive trajectories were modeled with piecewise linear splines, and rates of cognitive decline before, during, and after diagnosis were compared in the 2 groups. Data were analyzed from April 2022 to April 2024.

**Exposures:**

HMN diagnosis by Medicare diagnosis codes.

**Main Outcomes and Measures:**

Cognitive function was assessed by the Langa-Weir cognitive summary score from 1992 to 2020. Sociodemographic and health-related variables relevant to cognition were incorporated into propensity scores.

**Results:**

At baseline, there were 668 participants in the HMN cohort (mean [SD] age, 76.8 [7.6] years; 343 [51.3%] male; 72 [10.8%] Black, 33 [4.9%] Hispanic, and 585 [87.6%] White) and 1994 participants in the control cohort (mean [SD] age, 76.5 [7.3] years; 1020 [51.2%] male; 226 [11.3%] Black, 91 [4.6%] Hispanic, and 1726 [86.6%] White). The HMN cohort consisted predominantly of more indolent diagnoses, and only 96 patients (14.4%) received chemotherapy. Before and in the 2 years around the time of diagnosis, the HMN and control cohorts had similar rates of cognitive decline. At 1 year postdiagnosis and beyond, the rate of cognitive decline was slower in the HMN cohort (−0.18; 95% CI, −0.23 to –0.14) than in the control group (−0.24; 95% CI, −0.26 to –0.23) (*P* = .02), but this difference was no longer significant after accounting for the competing risk of death (HMN group, −0.27; 95% CI, −0.34 to –0.19; control group, −0.30; 95% CI, −0.33 to –0.27; *P* = .48).

**Conclusions and Relevance:**

In this cohort study of older adults, the HMN and matched noncancer control cohorts had similar rates of cognitive decline before, during, and after diagnosis after accounting for the competing risk of death.

## Key Points

**Question:**

Does cognitive decline accelerate in older adults after diagnosis with a hematologic malignant neoplasm compared with the cognitive trajectory seen in normal aging?

**Findings:**

In this cohort study, 668 older adults with hematologic malignant neoplasms and 1994 matched participants without cancer had similar rates of cognitive decline before diagnosis, surrounding the time of diagnosis, and after diagnosis when the competing risk of death was accounted for.

**Meaning:**

These findings suggest the cognitive trajectory of older adults diagnosed with hematologic malignant neoplasm parallels the trajectory seen in normal aging.

## Teaser

This cohort study evaluates cognitive trajectories and rates of cognitive decline before and after diagnosis of malignant neoplasm in older adults.

## Introduction

More people with hematologic malignant neoplasms (HMN) are surviving long-term with the development of effective, well-tolerated treatments.^1,2^ Many patients with HMN have the potential for cure or long-term disease control. In 2024, the 5-year relative survival for the 2 most common HMNs (non-Hodgkin lymphoma and leukemias) are 74.3% and 67.0%, respectively.^3^ Understanding the impact of HMN on long-term outcomes and quality of life in older survivors is increasingly important. Cognitive impairment affects quality of life and functional status in cancer survivors, and the risk of cognitive decline is an important consideration in treatment decisions for older adults with serious illnesses.^4,5^ Cognitive decline is often reported after cancer diagnosis or treatment. Unfortunately, the literature on cancer-related cognitive impairment (CRCI) in HMN is limited, with most research focused on solid tumors.^6,7,8,9,10,11,12,13^

HMN biology and treatments may impact cognition differently from solid tumors. First, HMN and its treatments cause more cytopenias, which are associated with increased fatigue, decreased physical activity, and cardiovascular risk, which may impact cognition.^14,15,16^ Second, since HMNs are systemic diseases affecting the immune and hematopoietic systems, their disease biology may influence cognition independent of treatment, as has been suggested in chronic lymphocytic leukemia and lymphomas.^17,18,19,20^ Lastly, unlike solid tumors, which in early stages may be treated with surgery or radiation, most HMNs are treated with systemic therapy, which is associated with higher CRCI risk.^8,13^ Improved understanding of what cognitive changes to expect in HMN survivors is important for patient counseling and management.

In general, the study of cognitive change in aging and cancer is fraught with methodological challenges. CRCI studies have found mixed results, likely reflecting diverse patient populations, methodologies, cognitive assessments, and choice of controls.^8,21,22,23^ Some studies have baseline pretreatment cognitive performance, but most studies do not have precancer cognitive trajectories. Many factors can influence cognition: age, comorbidities, genetics, health behaviors, functional status, cognitive reserve, and psychosocial factors.^6,10,11^ These factors are important to consider when evaluating CRCI so we understand how observed changes differ from what would be expected with normal aging.

The goal of this study was to model cognitive trajectories and compare rates of cognitive decline before, during, and after diagnosis in older adults diagnosed with HMN, compared with propensity score–matched participants without cancer. We hypothesized that the rate of cognitive decline would be faster after HMN diagnosis compared with normal aging.

## Methods

### Data Source

We used data from the Health and Retirement Study (HRS), which is a prospective longitudinal panel study surveying a population-representative sample of community-dwelling US adults since 1992.^24^ HRS participants undergo detailed interviews every 2 years from cohort entry at age 50 or older until death or dropout, with data collected about health, cognition, and more. HRS is sponsored by the National Institute on Aging and conducted by the University of Michigan. Informed consent and data collection were approved and overseen by the University of Michigan institutional review board. Ethical approval for data analysis for this study and waiver of patient consent due to use of deidentified data were obtained from the University of California, San Francisco Committee on Human Research. The study followed the Strengthening the Reporting of Observational Studies in Epidemiology (STROBE) guideline for reporting observational studies.

### Study Participants

To create the HMN cohort, we identified HRS participants who agreed to release their Medicare records (approximately 80% of cohort).^24^ Participants must have been diagnosed with an HMN from 1998 to 2016 and be older than 65 years when diagnosed. Participants were excluded if they had dementia at diagnosis, lacked an HRS interview within 2 years before diagnosis, or did not have any Langa-Weir cognitive scores during their follow-up. Dementia status at diagnosis was determined by the cognitive category assigned based on Langa-Weir cognitive scores or proxy cognitive scores from the HRS assessment immediately preceding diagnosis. Langa-Weir cognitive scores from 1992 to 1998 and 2016 to 2020 were included in the trajectory analysis if available.

To create the control cohort, we identified HRS participants who were propensity score–matched to an individual in the HMN cohort in the same HRS wave. Participants without cancer were excluded if they missed responding to the wave used for matching, were aged 65 years or younger at time of matching, had dementia at time of matching, had a diagnosis of any cancer (hematologic or solid) between 1998 and 2016, or did not have any Langa-Weir scores during their follow-up.

### Measures

Our exposure of interest was diagnosis with an HMN. Cancer diagnoses were defined by the *International Classification of Diseases, Ninth Revision *and *Tenth Revision *diagnosis codes in Medicare inpatient and outpatient billing files between 1998 and 2016, as primary or secondary diagnoses (eTable 1 in Supplement 1).^25,26^

### Outcome Variables

Our primary outcome was cognitive function as assessed by the Langa-Weir global cognitive summary score, which was developed and validated for identifying dementia in HRS and correctly identifies dementia in 52.18% and normal cognition in 87.17% of cases.^27^ The Langa-Weir is one of the standard cognitive assessments used extensively in many HRS studies.^28,29^ It is composed of 4 items: immediate 10-word recall (0-10), delayed 10-word recall (0-10), serial sevens to assess working memory (0-5), and backward counting to assess attention and processing speed (0-2). Scores range from 0 to 27 points, where 0 to 6 is dementia, 7 to 11 is cognitive impairment without dementia, and 12 to 27 is normal.

### Other Key Variables

Sociodemographic and health-related variables were selected a priori for incorporation into propensity scores. Values were drawn from the HRS interview within 2 years before diagnosis. When available, variables cleaned and processed by HRS or RAND were used.^30,31,32^ In total, 23 variables relevant to cognition were incorporated into propensity scores: sociodemographics (age, sex, self-reported race and ethnicity, education, marital status, and wealth), comorbidities (diabetes, heart disease, stroke, and depression), health behaviors (smoking, alcohol, physical activity, and body mass index), geriatric variables (activities of daily living, instrumental activities of daily living, hearing and/or vision impairment, and falls), and psychosocial variables (fatigue, sleep, and loneliness) (eTable 2 in Supplement 1). Reporting race and ethnicity in this study was mandated by the US National Institutes of Health, consistent with the Inclusion of Women, Minorities, and Children policy. Three variables (stress, discrimination, and apolipoprotein E genotype) were missing in more than 5% of the HMN cohort and thus were not included in propensity scores. Modal imputation was used for missing data in variables missing in 5% or fewer cases.

### Statistical Analysis

We used propensity score–based methods to evaluate the association between HMN diagnosis and cognitive outcomes. The propensity score was estimated using a logistic regression model that used multiple variables to estimate HMN diagnosis. To match participants with HMN to those without cancer, we first calculated propensity scores for eligible HMN participants using data from the HRS assessment immediately preceding diagnosis (baseline assessment). Then we calculated propensity scores for each potential eligible control in each HRS wave. We matched HMN participants to those without cancer in the same wave using greedy nearest-neighbor matching using a 1:3 ratio and calipers equal to 0.2 of the SD of the logit of the propensity score. We evaluated the quality of matching by comparing standardized mean differences between groups for each covariate.^33^

Time 0 for HMN participants was the date of diagnosis, which could be 0 to 730 days from their baseline assessment. A pseudo-time 0 was assigned for the control group based on their HMN match, such that pseudo-time 0 was the same number of days from the control’s matched baseline assessment. If the control died before pseudo-time 0, a second round of matching was performed to maximize the number of matches.

#### Model Selection

After trialing different ways of modeling cognitive trajectories, we compared 2 models with most face validity: a cubic spline with 4 knots (−1, −0.25, 0.25, and 1 year) and a piecewise linear spline with 2 knots (−1 and 1 year). The cubic and linear models had similar cognitive slopes, Akaike information criterion, and bayesian information criterion, with the linear model showing slightly better fit. The linear model also enabled direct comparison of slopes and CIs between cohorts. Thus, we chose the piecewise linear spline for our final analyses.

#### Sensitivity and Supplemental Analyses

There is potential bias due the competing risk of death if patients with cancer were more likely than those without to die or drop out, such that the fittest individuals who survived longer would be overrepresented in the postdiagnosis cognitive trajectory. Thus, we performed 2 sensitivity analyses. First, we applied inverse probability weighting (IPW) for mortality, which adjusts for the individual’s survival probability, such that cognitive data of those with a lower survival probability (eg, more similar to participants who died) were weighted more.^34^ Second, we repeated the analyses while limiting the analytic cohort to those who completed at least 2 HRS assessments before and 2 assessments after time 0. This approach ensured we were comparing participants who survived for at least 2 to 4 years after time 0 and had nonmissing HRS assessments before and after diagnosis to establish a cognitive trajectory.

As supplemental analysis, we repeated the analyses with survey weighting so the study sample more accurately represented the population of community-dwelling US adults. We did not use the weighted analysis as the primary approach since the analytic dataset represented only a small portion of the full HRS population, but it is still important to compare weighted and unweighted results.^35^ Statistical analyses were conducted using SAS version 9.4 (SAS Institute) and Stata version 18.0 (Stata Corp). Data were analyzed from April 2022 to April 2024.

## Results

### Participant Characteristics and Propensity Score-Matching

**Figure 1.**
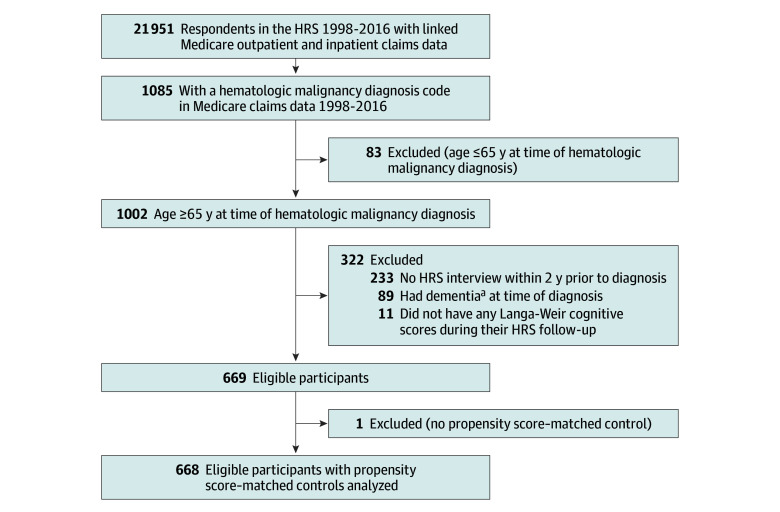
Study Cohort Creation HRS indicates Health and Retirement Study. ^a^Dementia at time of hematologic malignant neoplasm diagnosis determined by cognitive function category (dementia, cognitively impaired no dementia, normal) based on Langa-Weir cognitive scores and proxy cognitive scores in the HRS wave immediately preceding diagnosis.

At baseline, there were 668 participants in the HMN cohort (mean [SD] age, 76.8 [7.6] years; 343 [51.3%] male; 72 [10.8%] Black, 33 [4.9%] Hispanic, and 585 [87.6%] White) and 1994 participants in the control cohort (mean [SD] age, 76.5 [7.3] years; 1020 [51.2%] male; 226 [11.3%] Black, 91 [4.6%] Hispanic, and 1726 [86.6%] White). The mean (SD) follow-up from first to last HRS assessment evaluated was 13.0 (6.8) years in the HMN cohort and 16.2 (6.7) years in the control cohort. To define the HMN cohort, we identified 1085 HRS respondents with linked Medicare data who had an HMN diagnosis recorded between 1998 to 2016. Of these, we excluded 83 for being 65 years or younger at diagnosis, 89 for having dementia at diagnosis, and 244 for lacking adequate assessments for analysis, resulting in 669 eligible individuals. After propensity score–matching, 658 had 3 matches and 10 had 2 matches, resulting in 668 individuals in the HMN cohort and 1994 participants in the control cohort (Figure 1). The 244 individuals with an HMN diagnosis who were ineligible due to lacking adequate assessments were not significantly different from the HMN cohort in age, sex, race, or ethnicity but were more likely to have lower levels of education (χ^2^_3_ = 15.0; *P* = .002).

**Figure 2.**
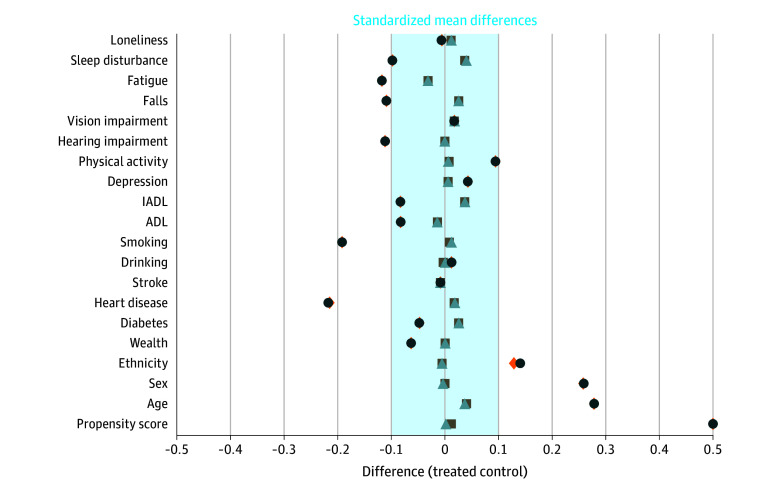
Standardized Mean Differences Before and After Propensity Score–Matching of Hematologic Malignant Neoplasm and Control Groups by Variables Relevant to Cognition All prematch differences are shown with dark blue circles. Region prematch differences are shown with orange diamonds. All matched differences are shown with gray squares. Weighted matched differences are shown with light blue triangles. Shaded area indicates negligible differences. Nonbinary variables included in matching but not shown: race, education, marital status, and body mass index. ADL indicates activities of daily living; IADL, instrumental  activities of daily living.

At baseline, 168 patients in the HMN cohort (25.1%) and 487 patients in the control cohort (24.4%) had cognitive impairment without dementia. Within the HMN cohort, the most common diagnoses were myeloproliferative neoplasm (127 patients [19.0%]), chronic leukemia (106 patients [15.9%]), and multiple myeloma (97 patients [14.5%]). Overall, 477 patients (71.4%) had diagnoses usually treated with chronic or intermittent therapy (eg, myelodysplastic syndrome, indolent lymphomas, or multiple myeloma), and 87 patients (13.0%) had aggressive diagnoses usually treated with curative intent therapy (eg, acute leukemias or aggressive lymphomas). Only 96 patients (14.4%) in the HMN group received chemotherapy. Baseline characteristics are described in Table 1. Matched cohorts were well-balanced on propensity score distribution and baseline characteristics. Standardized mean differences for all covariates were less than 0.1 (Figure 2).

**Table 1.** Baseline Characteristics of Hematologic Malignant Neoplasm (HMN) and Propensity Score–Matched Noncancer Control Cohorts

Characteristic	Patients, No. (%)		*P* value
	HMN cohort (n = 668)	Noncancer cohort (n = 1994)	
Sociodemographics			
Age, mean (SD), y	76.8 (7.6)	76.5 (7.3)	.39
Sex			
Female	325 (48.7)	974 (48.8)	.93
Male	343 (51.3)	1020 (51.2)	
Race			
Black/African American	72 (10.8)	226 (11.3)	.69
White	585 (87.6)	1726 (86.6)	
Other	11 (1.6)	42 (2.1)	
Ethnicity			
Hispanic	33 (4.9)	91 (4.6)	.69
Non-Hispanic	635 (95.1)	1903 (95.4)	
Education			
<High-school/GED	166 (24.9)	509 (25.5)	.96
High-school graduate	200 (29.9)	577 (28.9)	
Some college	157 (23.5)	473 (23.7)	
≥College	145 (21.7)	435 (21.8)	
Marital status			
Married/partnered	404 (60.5)	1199 (60.1)	.91
Separated/divorced	56 (8.4)	158 (7.9)	
Widowed	182 (27.2)	548 (27.5)	
Never married	26 (3.9)	89 (4.5)	
Wealth over median	333 (49.9)	1000 (50.2)	.89
Comorbidities			
Diabetes	160 (24.0)	493 (24.7)	.69
Heart disease	263 (39.4)	797 (40.0)	.78
Stroke	66 (9.9)	186 (9.3)	.67
Depression	76 (11.4)	227 (11.4)	.99
Health behaviors			
Smoking	430 (64.4)	1280 (64.2)	.93
Alcohol use	309 (46.3)	920 (46.1)	.96
Vigorous physical activity	153 (22.9)	467 (23.4)	.78
Body mass index^a^			
Underweight (<18.5)	9 (1.3)	27 (1.3)	.98
Normal (18.5-24.9)	243 (36.4)	743 (37.3)	
Overweight (25-29.9)	270 (40.4)	800 (40.1)	
Obese (≥30)	146 (21.9)	424 (21.3)	
Geriatric syndromes			
ADL, any difficulty	132 (19.8)	360 (18.1)	.33
IADL, any difficulty	113 (16.9)	346 (17.4)	.80
Cognitive impairment	168 (25.1)	487 (24.4)	.71
Hearing impairment	194 (29.0)	578 (29.0)	.98
Vision impairment	150 (22.5)	457 (22.9)	.80
Falls, any	249 (37.3)	760 (38.1)	.70
Psychosocial			
Fatigue	143 (21.4)	393 (19.7)	.34
Sleep disturbance	218 (32.6)	682 (34.2)	.46
Loneliness	111 (16.6)	338 (17.0)	.84
Disease/treatment^b^			
Myeloproliferative neoplasm	127 (19.0)	NA	NA
Chronic leukemia	106 (15.9)	NA	
Multiple myeloma	97 (14.5)	NA	
Myelodysplastic syndrome	78 (11.7)	NA	
Indolent lymphoma	69 (10.3)	NA	
Aggressive lymphoma	51 (7.6)	NA	
Acute leukemia	36 (5.4)	NA	
Other	104 (15.6)	NA	
Chemotherapy received	96 (14.4)	NA	
Time to treatment, median (IQR), d	177.5 (30-1025.5)	NA	

Abbreviations: ADL, activities of daily living; GED, General Education Development; IADL, instrumental activities of daily living; NA, not applicable.

^a^ Body mass index is calculated as weight in kilograms divided by height in meters squared.

^b^ Disease type, chemotherapy received, and time to treatment are not applicable to the noncancer control cohort and marked with NA.

### Cognitive Trajectories of HMN and Control Cohorts

**Figure 3.**
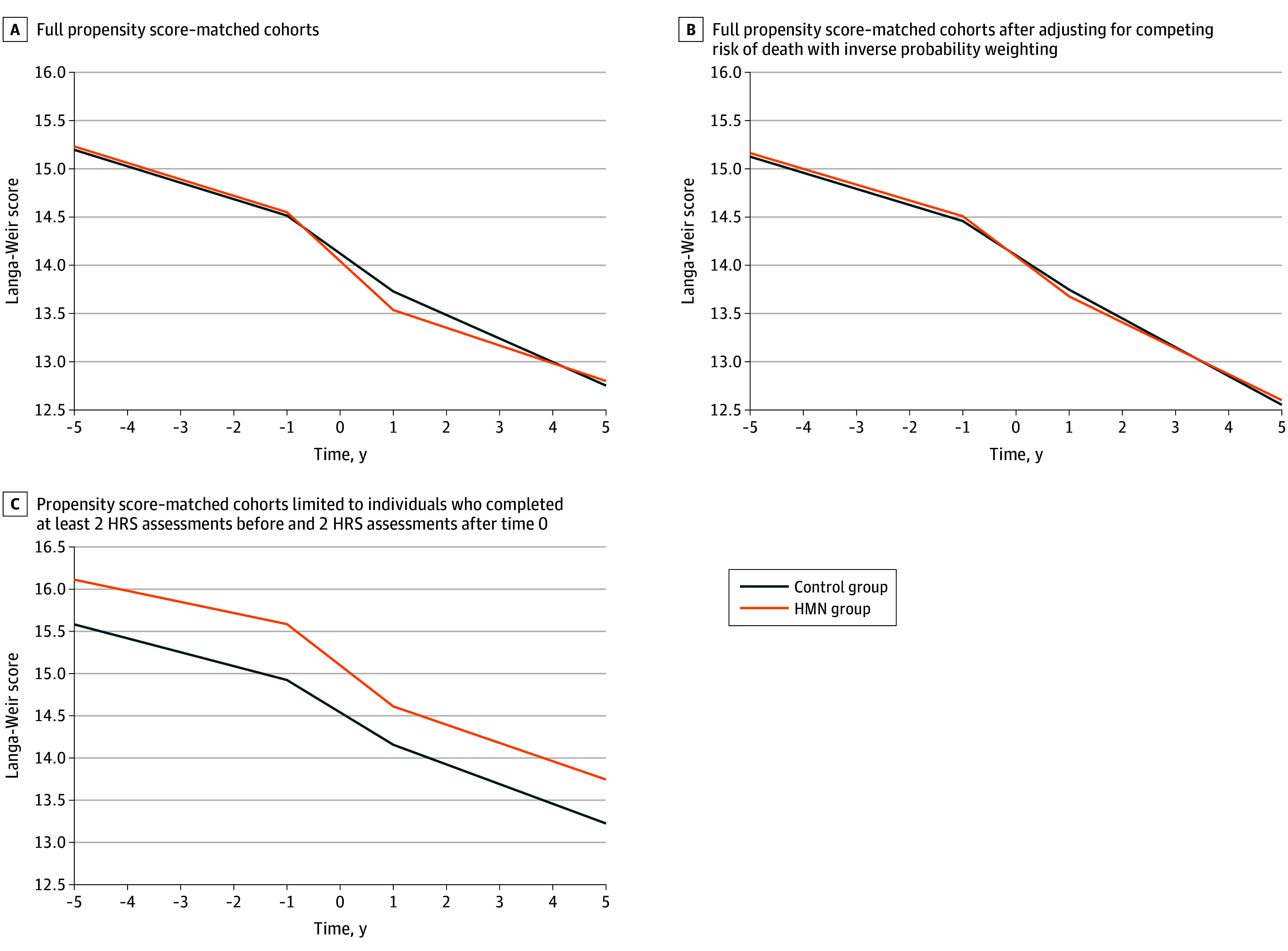
Trajectory of Langa-Weir Cognitive Summary Scores Over Time in the Hematologic Malignant Neoplasm (HMN) and Noncancer Control Groups Langa-Weir cognitive summary scores range from 0 to 27, with higher scores being better. Time 0 is the time of HMN diagnosis according to diagnosis codes from Medicare claims data. HRS indicates Health and Retirement Study.

Cognitive trajectories in the propensity-matched cohorts are shown in Figure 3A, and the corresponding Langa-Weir cognitive slopes are presented in Table 2. At baseline before time 0, similar rates of cognitive decline as measured by Langa-Weir score were seen in the HMN (−0.17; 95% CI, −0.19 to −0.15) and control (−0.17; 95% CI, −0.18 to −0.16) (*P* > .99) cohorts. In the 2 years peridiagnosis, the HMN group seemed to have a faster rate of cognitive decline (−0.51; 95% CI, −0.65 to –0.36) compared with the control group (−0.39; 95% CI, −0.46 to –0.32); however, this difference was not significant (*P* = .18). At 1 year postdiagnosis and beyond, the rate of cognitive decline was slower in the HMN cohort (−0.18; 95% CI, −0.23 to –0.14) than the control cohort (−0.24; 95% CI, −0.26 to –0.23; *P* = .02). For reference, a slope of −0.24 means that cognitive performance decreases by about 1 point in 4 years, which is the equivalent of missing 1 word on a 10-word recall or missing 1 subtraction on serial sevens.

**Table 2.** Change in Langa-Weir Cognitive Summary Score Modeled by a Linear Spline With Knots at 1 Year Before and 1 Year After Time 0

Cohort	Slope in Langa-Weir score (95% CI)		
	Before 1 y before time 0	During 1 y before to 1 y after time 0	Beyond 1 y after time 0
Full propensity score–matched cohorts			
HMN cohort (n = 668)	−0.17 (−0.19 to −0.15)	−0.51 (−0.65 to −0.36)	−0.18 (−0.23 to −0.14)
Noncancer cohort (n = 1994)	−0.17 (−0.18 to −0.16)	−0.39 (−0.46 to −0.32)	−0.24 (−0.26 to −0.23)
Between-group *P* value	>.99	.18	.02
Full propensity score–matched cohorts after adjusting for competing risk of death with IPW			
HMN cohort (n = 668)	−0.16 (−0.19 to −0.13)	−0.42 (−0.60 to −0.23)	−0.27 (−0.34 to −0.19)
Noncancer cohort (n = 1994)	−0.17 (−0.18 to −0.15)	−0.36 (−0.44 to −0.27)	−0.30 (−0.33 to −0.27)
Between-group *P* value	.85	.57	.48
Propensity score–matched cohorts limited to individuals who completed at least 2 HRS assessments before and 2 assessments after time 0			
HMN cohort (n = 668)	−0.13 (−0.16 to −0.10)	−0.49 (−0.67 to −0.30)	−0.22 (−0.27 to −0.16)
Noncancer cohort (n = 1994)	−0.16 (−0.19 to −0.14)	−0.38 (−0.50 to −0.26)	−0.23 (−0.26 to −0.20)
Between-group *P* value	.09	.36	.58

Abbreviations: HMN, hematologic malignant neoplasm; HRS, Health and Retirement Study; IPW, inverse probability weighting.

### Sensitivity and Supplemental Analyses

In both sensitivity analyses accounting for the competing risk of death, there were no significant differences in rates of cognitive decline between the 2 cohorts during any time period (Figure 3B and 3C; Table 2). Using IPW for mortality, rates of cognitive decline were similar in the HMN (−0.27; 95% CI, −0.34 to –0.19) and control cohorts (−0.30; 95% CI, −0.33 to –0.27). Limiting the analytic cohort to those with longer pretime and posttime 0 follow-up, rates of cognitive decline were also similar in the HMN (−0.22; 95% CI, −0.27 to –0.16) and control cohorts (−0.23; 95% CI, −0.26 to –0.20). Notably, with the latter approach, the HMN group had absolute Langa-Weir scores that were consistently higher than the control group (Figure 3C). Supplemental analysis using survey weights produced globally similar results (eTable 3 in Supplement 1).

## Discussion

Contrary to our hypothesis, the rate of cognitive decline in older adults after HMN diagnosis was not faster compared with normal aging. At baseline before and in the 2 years surrounding time 0, the HMN and control cohorts had similar rates of cognitive decline. Posttime 0, the rate of cognitive decline appeared slower in the HMN cohort than the control cohort, but this difference was no longer statistically significant after accounting for the competing risk of death.

Our results are concordant with other longitudinal cognitive studies in patients with cancer, which found slower or similar rates of cognitive decline in cancer survivors compared with participants without cancer. To our knowledge, no prior longitudinal studies with prediagnosis cognitive trajectories have focused on HMN. Ospina-Romero et al^36^ found that HRS participants aged 50 years or older with cancer had better memory function and slower memory decline after diagnosis compared with participants without cancer. To address survival bias, their sensitivity analysis in individuals with similar follow-up time yielded similar results. Wang et al^37^ found that a history of cancer did not exacerbate cognitive functioning in adults aged 65 years or older in HRS, and adults aged 75 to 84 years had slower cognitive decline compared with participants without cancer. A meta-analysis^23^ of longitudinal studies investigating cognitive performance in adults treated with chemotherapy found that patients’ performance improved from baseline to follow-up more than the improvement seen in the control group. In a population-based study, Anstey et al^38,39^ found that those aged 60 years and older with cancer who did not receive chemotherapy had similar cognitive performance as participants without cancer.

The counterintuitive finding of slower cognitive decline in cancer survivors compared with participants without cancer may support the proposed inverse relationship between cancer and Alzheimer disease, where factors related to carcinogenesis may be neuroprotective.^40,41,42,43,44,45^ Another possible explanation is posttraumatic growth among cancer survivors, which posits that cancer survivors may be incentivized to adopt lifestyle changes beneficial to cognitive functioning.^46,47^ Alternatively, this paradoxical inverse relationship may be due to methodological biases, such as competing risks, survival bias, or handling of potential confounders.^48,49,50^

In our study accounting for the competing risk of death, after 1 year or more postdiagnosis, the HMN group demonstrated similar rates of cognitive decline as matched participants without cancer. The postdiagnosis rate of cognitive decline in the HMN group was steeper with IPW (−0.27) than in the primary analysis (−0.18), consistent with the concern that those with a higher probability of mortality (therefore upweighted with IPW) exhibit faster cognitive decline that would not be reflected in the primary analysis. These results suggest that the competing risk of death does bias results toward better cognitive performance in survivors.

It is important to acknowledge that our HMN cohort is composed predominantly of indolent diseases, and only 14.4% received chemotherapy. Thus, these cognitive trajectories represent primarily indolent diagnoses which may not require therapy for years. Cognitive trajectories may look different if the cohort included mostly aggressive diagnoses or those treated with chemotherapy. One future direction of this work is to describe patterns of cognitive decline in different disease and treatment subgroups while accounting for potential confounders for chemotherapy receipt.

### Strengths and Limitations

Strengths of this study include the focus on HMN, a large population-representative sample, longitudinal cognitive data that precede cancer diagnosis, use of Medicare claims data rather than self-report of cancer to minimize misclassification, and accounting for the competing risk of death. This study has several limitations. First, the trajectories included only Langa-Weir scores based on assessments completed by the participant. Participants who only had proxy report for cognition were not included in the trajectory analysis given the small proportion (<5% at baseline) and lack of a validated way to map proxy scores to Langa-Weir scores, although proxy scores were used to determine baseline dementia status. Second, we acknowledge limitations of the Langa-Weir, which only correctly identifies dementia in 52.18% of cases and does not cover all cognitive domains. However, it is useful for evaluating change over time and covers most domains implicated in CRCI (eg, learning and memory, attention, working memory, processing speed). Third, different HMN diagnoses exhibit heterogenous clinical courses and are managed differently, and we do not account for this heterogeneity due to the small resulting sample sizes. The CRCI literature suggests that both disease and treatment may impact cognition, and thus different disease or treatment subgroups may exhibit different cognitive trajectories, which is an important area for future research. Fourth, with propensity score-based matching, there is the possibility of unmeasured confounding. However, we matched on a very broad set of variables and our prediagnosis cognitive trajectories are essentially identical between the 2 groups, suggesting that they are well-matched on risk factors for cognition. Fifth, our cohorts included individuals who completed adequate assessments for analysis and thus represent a highly selected group, which may impact generalizability to the standard population. Additionally, our selection method of participants without cancer may miss individuals cured of cancer before 1998, which would not be captured by diagnosis code review during 1998 to 2016.

## Conclusions

In conclusion, after accounting for the competing risk of death, the cognitive trajectories of older adults with HMN paralleled that of normal aging. The competing risk of death should be accounted for in longitudinal studies of cognition in older adults with cancer to avoid potential bias. Despite frequent concerns about CRCI, we did not find evidence that HMN diagnosis accelerates cognitive decline more than normal aging, which can be helpful for counseling patients on what to expect cognitively after diagnosis. Future directions include identifying predictors of clinically meaningful cognitive decline and exploring whether different diagnosis and treatment types will result in heterogeneous cognitive trajectories.

## Notes

Supplement 1. **eTable 1. **Definition of Cancer-Related Exposure and Covariates **eTable 2. **Sociodemographic and Health-Related Variables Included in Propensity Score Model **eTable 3. **Change in Langa-Weir Cognitive Summary Score in Hematologic Malignant Neoplasm and Propensity Score–Matched Noncancer Control Cohorts in Models With Survey Weighting

Supplement 2.
Data Sharing Statement
