# Supplementary material for: Cognitive Trajectories in Older Adults Diagnosed With Hematologic Malignant Neoplasms
Source: JAMA Netw Open. 2024 Aug 30;7(8):e2431057. doi: 10.1001/jamanetworkopen.2024.31057 (PMC11365001; doi:10.1001/jamanetworkopen.2024.31057)
Supplement: Supplement 1. — eTable 1. Definition of Cancer-Related Exposure and Covariates eTable 2. Sociodemographic and Health-Related Variables Included in Propensity Score Model eTable 3. Change in Langa-Weir Cognitive Summary Score in Hematologic Malignant Neoplasm and Propensity Score–Matched Noncancer Control Cohorts in Models With Survey Weighting [file jamanetwopen-e2431057-s001.pdf]

Supplemental Online Content

Huang L-W, Shi Y, Boscardin WJ, Steinman MA. Cognitive trajectories in older adults diagnosed with hematologic malignant neoplasms. *JAMA Netw Open*. 2024;7(8):e2431057. doi:10.1001/jamanetworkopen.2024.31057

eTable 1. Definition of Cancer-Related Exposure and Covariates

eTable 2. Sociodemographic and Health-Related Variables Included in Propensity Score Model

eTable 3. Change in Langa-Weir Cognitive Summary Score in Hematologic Malignant Neoplasm and Propensity Score-Matched Noncancer Control Cohorts in Models With Survey Weighting

This supplemental material has been provided by the authors to give readers additional information about their work.

© 2024 Huang L-W et al. *JAMA Network Open.*

eTable 1. Definition of Cancer-Related Exposure and Covariates

Variable	ICD-9 code	ICD-10 code
Hematologic malignant neoplasms	200-208, 238.4-238.7, 273.3, 284.0, 289.83	C81-C96, D45-D47.1, D47.3-D47.4, D47.Z, D61.0, D61.3, D71.81
Non-hematologic cancers	140.00–199.2, 209.00-209.36, 209.7	C00-C75, C7A, C7B, C76-C80
Chemotherapy administration	V58.11, V58.12	Z51.11, Z51.12
Hematopoietic cell transplant	V42.81, V42.82	Z94.81, Z94.84

ICD=International Classification of Diseases.

© 2024 Huang L-W et al. *JAMA Network Open.*

eTable 2. Sociodemographic and Health-Related Variables Included in Propensity Score Model

	Variable	Variable source (variable name)	Description
Socio-demographics	age	Medicare, RAND HRS (RWAGEY_M)	Hematologic malignant neoplasm cohort: Age at time of ICD9/10 code for HM cohort. Control cohort: age corresponding to the wave on which they are matched.
	sex	RAND HRS (RAGENDER)	Biological sex assigned at birth. 1=male, 2=female.
	race	RAND HRS (RARACEM)	Race. 1=White/Caucasian, 2=Black/African American, 3=other.
	ethnicity	RAND HRS (RAHISPAN)	Ethnicity, whether Hispanic. 0=not Hispanic, 1=Hispanic.
	education	RAND HRS (RAEDUC)	Categorical education. 0=less than high school, GED; 1=high school graduate; 2=some college, 3=college and above.
	marital status	RAND HRS (RwMSTAT)	Marital status. 1=married, married spouse absence, partnered; 2=separated, divorced; 3=widowed; 4=never married.
	wealth	RAND HRS (HwATOTB)	Net value of total wealth calculated as the sum of all wealth components less all debt. Dichotomized at median $214,000. 0=at or below weighted median, 1=above weighted median.
Comorbidities	diabetes	RAND HRS (RwDIABE)	Whether or not a doctor has ever told the Respondent s/he had diabetes. 0=no, 1=yes.
	heart disease	RAND HRS (RwHEARTE)	Whether or not a doctor has ever told the Respondent s/he had heart disease. 0=no, 1=yes.
	stroke	RAND HRS (RwSTROKE)	Whether or not a doctor has ever told the Respondent s/he had a stroke. 0=no, 1=yes.
	depression	RAND HRS (RwCESD)	Measure of depression over the week prior to interview, using the 8-item Center for Epidemiologic Studies Depression scale. 0=score <4 not depressed, 1=score ≥4 depressed.
Health behaviors	smoking	RAND HRS (RwSMOKEV)	Whether the Respondent ever smoked cigarettes. 0=no, 1=yes.
	alcohol use	RAND HRS (RwDRINK)	Whether the respondent ever drinks any alcohol. 0=no, 1=yes.
	physical activity	RAND HRS (RwVIGACT for waves 1-6, RwVGACTX for waves 7-14)	Vigorous physical activity 3+/wk. 0=no, 1=yes.<br><br>Frequency of vigorous physical activity. 0=never, 1-3/month, 1/week; 1=every day, >1/week.
	BMI	RAND HRS (RwBMI)	Self-reported height & weight used to calculate body mass index kg/m2.
Geriatric syndromes	ADL impairment	RAND HRS (RwADLA)	Sum of ADLs where respondents report any difficulty: bathe, dress, eat, get in/out of bed, walking across a room. 0=no difficulty (sum 0), 1=any difficulty (sum 1-5).
	IADL impairment	RAND HRS (RwIADL5A)	Sum of IADLs where respondents report any difficulty: using the phone, managing money, taking medications, shopping for groceries, preparing hot meals. 0=no difficulty (sum 0), 1=any difficulty (sum 1-5).

© 2024 Huang L-W et al. *JAMA Network Open.*

eTable 2. Sociodemographic and Health-Related Variables Included in Propensity Score Model (continued)

	Variable	Variable source (variable name)	Description
Geriatric syndromes	Hearing impairment	Harmonized HRS C (RwHEARING)	Respondent's self-rated hearing. 0=excellent, very good, good; 1=fair, poor.
	vision impairment	Harmonized HRS C (RwSIGHT)	Respondent's self-rated eyesight. 0=excellent, very good, good; 1=fair, poor, blind.
	falls	Harmonized HRS C (RwFALL)	Whether the respondent has fallen down in the last 2 years. 0=no, 1=yes.
Psycho-social	fatigue	Harmonized HRS C (RwFATIGUE)	Whether the respondent has experienced severe fatigue or exhaustion. 0=no, 1=yes.
	sleep disturbance	HRS raw data (E987 for wave 3, F1495 for wave 4, G1671 for wave 5, *D112 for waves 6-14)	Sleep was restless in the past week. 0=no, 1=yes.
	loneliness	HRS raw data (E989 for wave 3, F1497 for wave 4, G1673 for wave 5, *D114 for waves 6-14)	Felt lonely in the past week. 0=no, 1=yes.

HRS=Health and Retirement Study, ICD=International Classification of Diseases, GED=General Education Development, BMI=body mass index, ADL=activities of daily living, IADL=instrumental activities of daily living.

© 2024 Huang L-W et al. *JAMA Network Open.*

eTable 3. Change in Langa-Weir Cognitive Summary Score in Hematologic Malignant Neoplasm and Propensity Score-Matched Noncancer Control Cohorts in Models With Survey Weighting

	**Slope in Langa-Weir score (95% CI)**		
	Prior to 1 year before time 0	During 1 year before to 1 year after time 0	Beyond 1 year after time 0
**A. Full propensity score-matched cohorts with survey weighting**			
Hematologic malignant neoplasm (n=668)	-0.18 (-0.22, -0.15)	-0.42 (-0.66, -0.17)	-0.19 (-0.25, -0.13)
Non-cancer control (n=1994)	-0.17 (-0.18, -0.15)	-0.38 (-0.46, -0.29)	-0.25 (-0.28, -0.22)
Between group p-value	*0.38*	*0.76*	*0.08*
**B. Full propensity score-matched cohorts after adjusting for competing risk of death with IPW and with survey weighting**			
Hematologic malignant neoplasm (n=668)	-0.18 (-0.22, -0.14)	-0.36 (-0.60, -0.13)	-0.25 (-0.32, -0.17)
Non-cancer control (n=1994)	-0.17 (-0.19, -0.15)	-0.34 (-0.41, -0.26)	-0.30 (-0.33, -0.26)
Between group p-value	*0.56*	*0.82*	*0.21*
**C. Propensity score-matched cohorts limited to individuals who completed at least 2 HRS assessment before and 2 assessments after time 0, with survey weighting**			
Hematologic malignant neoplasm (n=264)	-0.15 (-0.20, -0.09)	-0.38 (-0.66, -0.10)	-0.22 (-0.29, -0.16)
Non-cancer control (n=585)	-0.16 (-0.19, -0.13)	-0.36 (-0.51, -0.21)	-0.24 (-0.28, -0.20)
Between group p-value	*0.60*	*0.92*	*0.70*

CI=confidence interval, IPW=inverse probability weighting, HRS=Health and Retirement Study.

© 2024 Huang L-W et al. *JAMA Network Open.*
