# Supplementary material for: Cognitive Trajectories in Older Adults Diagnosed With Hematologic Malignant Neoplasms
Source: JAMA Netw Open. 2024 Aug 30;7(8):e2431057. doi: 10.1001/jamanetworkopen.2024.31057 (PMC11365001; doi:10.1001/jamanetworkopen.2024.31057)
Supplement: Supplement 2. — Data Sharing Statement [file jamanetwopen-e2431057-s002.pdf]

Data Sharing Statement

Huang. Cognitive Trajectories in Older Adults Diagnosed With Hematologic Malignant Neoplasms. *JAMA Netw Open*. Published August 30, 2024.
doi:10.1001/jamanetworkopen.2024.31057

Data
Data available: No

Additional Information
Explanation for why data not available: The deidentified patient data used in this study is available through the Health and Retirement Study's public use datasets.
